# Supplementary material for: Medial prefrontal cortex encodes but is not required to generate goal-directed actions under threat
Source: eLife. 2026 Jul 8;15:RP110964. doi: 10.7554/eLife.110964 (PMC13345635; doi:10.7554/eLife.110964)
Supplement: Peer review history

# Editor report: eLife Assessment

**DOI:** 10.7554/eLife.110964.3.sa0

**Authors:** Laura A Bradfield

**Affiliations:** The University of Sydney Australia

**Keywords:** Compelling, Important

This **important** study employed a multi-stage behavioural paradigm of increasing cognitive complexity to investigate the role of inhibitory interneurons in the medial prefrontal cortex (mPFC) in avoidance behaviour in mice. The authors used imaging and optogenetic techniques combined with this behavioural task to show that mPFC interneurons are necessary for encoding but not executing avoidance under threat. The evidence supporting these claims is **compelling**, and findings will be of interest to researchers in behavioural and systems neurosciences.

# Referee report: Reviewer #1 (Public review)

**DOI:** 10.7554/eLife.110964.3.sa1

Summary:

This study investigates the role of the medial prefrontal cortex (mPFC) in generating goal-directed actions under threat, using a progressive behavioral paradigm, neural recordings, and optogenetic inhibition in mice. The authors demonstrate that while mPFC GABAergic neurons strongly encode cues, actions, and errors, particularly under high cognitive demand, this neural activity is not causally required for executing avoidance behaviors. By rigorously controlling for movement and arousal, the researchers found that much of the observed mPFC signaling actually reflects baseline behavioral states rather than the generation of the actions themselves. This dissociation between encoding and causality challenges traditional views of mPFC as an executive controller of action and provides a nuanced understanding of its role in evaluative and contextual processing.

Strengths:

The behavioral paradigm employed in this study is one of its greatest strengths, offering a rigorous, progressive, and well-controlled framework to dissect the neural mechanisms underlying avoidance under threat. This three-phase task design is particularly well-suited to tease apart the contributions of learning, discrimination, and cognitive load to both behavior and neural activity.

By tracking movement (speed, rotations) and including it as a covariate in statistical models, the authors also underscore the need to control for movement and baseline activity when interpreting cortical signals, which is relevant for all studies of brain-behavior relationships, ensuring that behavioral changes are not due to general arousal or motor activity.

Finally, the study combines multiple advanced techniques-fiber photometry, single-cell calcium imaging (miniscopes), and two distinct optogenetic inhibition methods-to provide a comprehensive look at both neural encoding and causal necessity.

Comments on revised version.

The authors adequately addressed all of the reviewers' comments and made great improvements to the manuscript, particularly enhancing the methods and figures to significantly improve clarity and readability.

# Referee report: Reviewer #2 (Public review)

**DOI:** 10.7554/eLife.110964.3.sa2

Summary:

The manuscript by Sajid et al. describes a comprehensive behavioral, imaging and optogenetic dataset investigating the role of the mPFC in avoidance and escape behaviors. Although many movement- and task-related variables are encoded by mPFC GABAergic neurons, the main conclusion is that they are unlikely to control behavioral output.

Strengths:

The manuscript is generally well executed and plausible in its conclusions. It provides an alternative viewpoint to many articles describing the involvement of mPFC to behavior, based on a complex multi-stage behavioral paradigm acquired and analyzed in an unbiased way.

Weaknesses:

This reviewer sees two weaknesses.

(1) In some cases, the explained variance, marginal and conditional, is low, suggesting the models only modestly capture the complexity in the data.

(2) The manuscript is challenging to read due to the comprehensive and unbiased presentation style.

Comments on revised version.

The authors did a good job at addressing the reviewers' comments. One minor additional suggestion is to add references for the statement in the last paragraph of the discussion for the mPFC lesion studies.

# Author comment: Author response

**DOI:** 10.7554/eLife.110964.3.sa3

**Authors:** Sarmad Sajid, Ji Zhou, Manuel A Castro-Alamancos

**Affiliations:** UConn Health Farmington United States; UConn Health Farmington United States; UConn Health Farmington United States

The following is the authors’ response to the original reviews.

**Public Reviews:**

**Reviewer #1 (Public review):**

The authors conclude that mPFC is not required for avoidance, based on the minimal behavioral effects of optogenetic inhibition. While this interpretation is supported by the data, the choice of viral constructs could lead to an underestimation of the mPFC's role for other reasons. First, the choice of viral constructs could lead to an underestimation of the mPFC's role for several reasons. Specifically, the efficacy of eArch3.0 inhibition was not verified beyond histology, and its non-cell-type-specific nature could lead to disinhibition or compensatory activity in downstream regions. Although the authors' use of visual cortex (VI) inhibition as a control suggests that broad cortical inhibition does not impair avoidance, subcortical compensation cannot be ruled out. Additionally, Vgat-ChR2 targets only GABAergic neurons, potentially missing glutamatergic contributions. Addressing these limitations in the Discussion section would strengthen the manuscript.

We thank the reviewer for these points. First, although we did not perform direct electrophysiological verification of eArch3.0 efficacy in mPFC in the present study, this construct has been extensively validated in prior work and is widely used to produce robust neuronal inhibition. In our experiments, the lack of behavioral effect with eArch3.0 inhibition converged with the results obtained using the independent Vgat-ChR2 approach, which we directly validated, supporting the conclusion that mPFC inhibition does not impair avoidance under these conditions. Our results are also consistent with previous studies showing that mPFC lesions do not impair avoidance behavior.

Second, we agree that manipulating mPFC activity will necessarily influence downstream circuits, including subcortical regions, given the interconnected nature of these networks. Our goal was to test whether inhibiting mPFC activity alters avoidance behavior, not to isolate it from its targets. In this context, the absence of behavioral effects indicates that avoidance behavior can be supported without mPFC activity. While compensation is always a possibility, this usually reveals some impairment while compensation occurs, but we did not observe those effects. Our results are consistent with the idea that subcortical circuits normally mediate these behaviors.

Finally, regarding Vgat-ChR2, activating GABAergic neurons is a well-established approach to suppress cortical activity, as these interneurons provide strong inhibition onto local glutamatergic neurons. Thus, this manipulation is expected to broadly reduce excitatory output in cortex. Indeed, the robust suppression of cortical activity we observed with GABAergic activation makes it unlikely that major glutamatergic contributions were missed.

These points are in the paper, including the Discussion.

**Reviewer #2 (Public review):**

(1) There are few details on the linear mixed models in the methods. This section could be improved by including a mathematical description. More importantly, the reader never learns how accurately the models capture the data. Given that most conclusions rely on the models, it seems central to address this point carefully. For example, what is the explained variance, marginal, and conditional? Were the nested models compared to non-nested ones (e.g., AIC), what are the specific outputs of the likelihood ratio tests briefly mentioned in the methods?

Model structure was defined a priori by the experimental design and hypotheses rather than selected through model comparison, but we verified the contribution of key model components (e.g., covariates, interactions, and random effects) using likelihood ratio tests comparing models. Regarding model performance, we now report for each model the marginal and conditional R^2^ values (Nakagawa), which quantify variance explained by fixed effects alone and by the full mixed model including random effects. In addition, likelihood ratio test results for all fixed effects and interactions (χ^2^ statistics) were already reported in the manuscript.

(2) For several figures, there is a disconnect with the main text, in the sense that it is difficult to understand how statements in the main text connect with specific figure panels or bars in their graphs. This is particularly the case for the most complex figures, e.g., Figures 3, 4, and their supplements. It would be beneficial to introduce subfigure labels (A1, etc) and state explicitly in the main text what figure panel is described (in parentheses). Alternatively, breakdown the figures into multiple ones, decreasing ambiguity. This is important because it will help the reader better assess the strength of the results.

We have significantly revised the manuscript to reduce ambiguity and thank the reviewer for each of their (28) requests, which we have implemented in full. We also added additional figure references to the Results to assist with readability. This has significantly improved clarity and readability.

(3) It does not appear that the code and data used to produce the figures are made available. That would be very beneficial, given the complexity of the analysis and dataset collection procedures. It would also help readers better understand the results and probe their validity.

As usual, we will share the full dataset in the VOR at Dryad after the revision is completed.

**Reviewer #3 (Public review):**

The main weakness, in my view, lies in the Results section. In the figures, the authors do not present any raw data, and the plots are shown as mean {plus minus} SEM without displaying the distribution of individual data points.

We thank the reviewer for the recommendations. Individual data points are shown where appropriate (e.g., Fig. 1). However, most of our analyses involve repeated-measures, hierarchical data with multiple levels (cells and sessions nested within animals), where simple point overlays can be misleading or difficult to interpret without explicit linking across levels. We therefore use mean ± SEM visualizations for clarity in these summary figures, while preserving the full hierarchical structure in the statistical analysis through mixed-effects models. All data will be made available in the VOR to allow full inspection of the underlying distributions.

It is both a strength and a weakness that the authors do not attempt to guide the reader through the Results section and instead present the findings with very little emphasis on the key outcomes of the GLM. While this approach is arguably the most transparent way to report results, it also makes the section quite difficult to follow and may discourage readers.

I would recommend rewriting the Results section to make it more accessible to a broader audience. A similar issue applies to the figures: presenting all plots reflects a commendable commitment to transparency, but it would greatly benefit from a clearer narrative. As it stands, it is difficult to grasp the message of each figure by simply browsing through them.

The full description (complexity) of the models is entirely in the legends and supplemental figures. This was done to make the results easier to follow. We have made all the changes noted above to facilitate readability while assuring there is enough transparency to assess the data. We think readability has significantly improved.

**Recommendations for the authors:**

**Reviewer #2 (Recommendations for the authors):**

Below are a few specific suggestions related to the main weaknesses mentioned above.

(1) P4 L9: The sentence starting with "However, most ..." sounds more like a statement than a contrast with the previous sentence. Therefore, please delete "However" and please add references to justify the statement.

Done.

(2) P8: Definition of movement peaks. It would be great to have three videos illustrating the mouse behavior in the three different movement peaks. This would allow the reader to better understand the differences between no peaks 3 sec prior, more than 5 seconds, and one example that does not fit these two categories. In addition, what percentage of all peaks to the no peaks 3 sec prior and more than 5 sec represent?

We added the percentages. The “3 sec prior” represent ~23% and the “5 sec” represent ~31%. However, we do not think adding a single video of one movement per these 3 cases would be useful as the dataset is composed of thousands of these movements.

(3) P8: Last paragraph. When you state that you performed a linear fit between DF/F and movement, do you mean speed? In addition, the statement "integrating both signals over a 200 ms window" is incomplete. How is the window selected? Is the window 200 ms around movement onset or movement peak speed?

Yes, the movement variable used in the linear fit corresponds to speed. Regarding the 200 ms window, this analysis does not focus on specific behavioral events such as movement onset or peak speed. Instead, both ΔF/F and speed signals were segmented into consecutive 200 ms windows across the entire recording session, and the linear relationship was computed across these paired segments. Thus, the analysis captures the overall relationship between neural activity and ongoing movement, rather than eventaligned dynamics. We have revised the text to clarify both the use of speed and the implementation of the 200 ms window.

(4) P14: Discussion of AA19 and AA39 tasks: It would be helpful to clearly specify what percentage of actions you would expect given no learning, is it the 23% action dashed line indicated in the top panel of Figure 2B?

The expected percentage of actions under no learning is not fixed, as it depends on the rate of spontaneous (non–cue-driven) crossings. In these tasks, we estimate this baseline using behavior during the noUS condition, where the action rate is ~23% (Fig. 2B). In the AA19 and especially AA39 tasks, this baseline decreases because spontaneous inter-trial crossings (ITCs) are progressively reduced, leading to lower expected action rates under no-learning conditions. Thus, the 23% baseline derived from noUS is lower in the AA19/39 tasks. In other studies, we explicitly included NoCS (no-cue) trials to estimate chance performance; however, in the present design we rely on the noUS baseline and the observed changes in ITC rate. We have clarified this point in the text.

(5) P15 L2: "Considering tone intensity (Fig. 2B), CS1 avoids latencies increased at medium and high intensities but not a low intensity." This is confusing. Are you referring to the AA39 triangles under CS1 in the middle panel, left? They are all above the dashed reference line. So the plot seems to contradict the statement. If you are referring to AA19, the red dots also seem to show the opposite of the statement.

The dashed reference line reflects latency during the noUS condition and is included for visual reference; however, these values are not directly comparable to those in the AA tasks, as noUS latencies are largely unconstrained and reflect baseline behavior rather than learned responding. The statement in the text refers specifically to changes across AA conditions, consistent with our analysis approach throughout the manuscript, where values are compared to the immediately preceding condition. In this case, we are referring to AA39 (triangles) relative to AA19 (circles). Under this comparison, CS1 avoidance latencies increase at medium and high intensities, but not at low intensity, consistent with the statistical contrasts. We have revised the text to clarify the points.

(6) P17: "Movement and neural measures subtract the baseline from the other three windows at a trial level." Do you mean to say that for each measure, the baseline was subtracted? How is baseline defined (over which time window)?

The baseline is defined in that same paragraph as the −0.5 to 0 s pre-CS window. To improve clarity, we have revised the text to explicitly restate this definition in the sentence describing baseline subtraction.

(7) P17: "Fig. 2-Supplement 2A,B shows model-derived marginal means of movement averaged across tone intensities." Some explanation needs to be provided, since the previous figures show a dependence of behavior on tone intensity. Are you doing this based on Fig. 2-S1?

Yes, these results are derived from the same model of the full data shown in Fig. 2–S1. In this particular analysis, tone intensity was included in the model but not retained when computing marginal means and contrasts, effectively averaging across intensity levels. The rationale for this approach is that tone intensity was primarily used to increase behavioral variability, particularly error rates, which are otherwise low in this task. Averaging across intensity therefore improves statistical power and allows us to more clearly isolate the effects of the primary factors of interest. We have clarified this point in the text.

(8) P18: "Orienting magnitude was strongly dependent on tone intensity...". However, in Figure 2-S2, there is no information about tone intensity. So how is the reader supposed to see this? Same issue on P19 when discussing the action window. Generally, the description of Figure 2-S1 and S2 is difficult to follow and should be improved. It is not clear that all panels are referred to in the text.

We have revised the start of the Movement section to clarify how tone intensity is treated across analyses and figures. Specifically, tone intensity is included as a factor in all statistical models; however, for clarity of presentation, it is sometimes collapsed in figures to reduce dimensionality and to emphasize other task-related factors. This manipulation was introduced primarily to increase behavioral variability (particularly error rates), thereby improving sensitivity for estimating the effects of the other task variables.

We have also clarified when we reference Fig. 2–S2 legend that, although intensity is not displayed in the figure for visualization purposes, it is included in the underlying model and its effects are reported in the supplement.

(9) P22, 23: Windows are mentioned, but not defined or indicated in figures.

We have clarified in the text that the same time windows defined for movement analyses (baseline, orienting, action, and from-action) were also used for the neural analyses.

(10) P22: "Covariates were standardized within each window so that estimated marginal means reflected ΔF/F at average covariate values." It is unclear what was done exactly. What do you mean by "standardized"? Maybe give an example here and elaborate in the methods.

By “standardized within each window,” we mean that covariates were z-scored within each analysis window (i.e., each covariate was transformed to have a mean of 0 and a standard deviation of 1 within that window). This ensures that estimated marginal means correspond to ΔF/F evaluated at the average covariate values within each window. We have clarified this in the Methods and Results.

(11) P24-25: Indicating spurious action on Figure 3-S2 (and in Figure 3) would help the reader follow the argument in the main text.

We clarified this in the legends by indicating that actions not classified as AA, PA, Escape, or PA Error are spurious actions.

(12) P25: "After controlling for ..., but this includes the effects of aversive stimulation." The second part of this sentence was not clear.

We have clarified this sentence to indicate that avoidance errors are followed by aversive stimulation (i.e., errors are punished).

(13) P34L3: "Classs" -> "Class".

Fixed.

(14) P42 top paragraph: There are two references to Figure 5-S1 panel D, but there is no panel D on the figure.

Fixed.

(15) P57: The sentence starting with "Random effects were specified ..." is very difficult to follow.

We have revised this sentence to improve clarity by separating the description of the random-effects structure from the model syntax.

(16) P57: The windows analyzed are finally defined at the bottom of this page. The information also needs to be included early in the results to improve comprehension.

This is now included in the main text when windows are first used in the movement section.

(17) P58: Several R packages are mentioned by name, but without specifying that they are R packages, which would facilitate reading.

We added R.

(18) P58 top paragraph: "Tuckey's correction", do you mean "Tukey's HSD test"?

We thank the reviewer for noting this. We used Holm-adjusted p-values for multiple comparisons (as implemented in emmeans) and have revised the text.

(19) P63: "features extracted from F/F" do you mean "DF/F"?

Yes, fixed.

(20) Figure 1B speed plots: it is not possible to visualize the lines at the movement peak because they overlap completely. You can either add an inset on the left of the peak (for each panel), magnifying that region, or play with the transparency of the traces to improve visibility. There is a similar issue in Figure 5A, B. (Alternatively, if it is not possible to solve the issue graphically, explicitly state that traces overlap.)

We have fixed this by making some traces dashed in Figure1 and 1-S1, which reveals the underlying traces. We also stated that the peak speed completely overlaps. In Figure 5, we stated that traces overlap as expected; transparency or dashing does not work well with the colors used in Figure 5 and in fact the overlap emphasizes the similarity of the movements.

(21) Legend 1A: abbreviation CCF not defined. Is it anterior to the left? Abbreviation WM not defined. The right panels are unclear. The legend states that they show a schematic of the location of the optical fibers, but that was not clear. Do the dots indicate the location of the fibers? Is the green region indicative of V1? Same for dark gray in the mPFC panel. What are the lighter grey regions and the blue region? Does 'lateral' mean 'lateral from midline'? Please clarify these points.

CCF is defined in Methods, and the typesetting process will adjust abbreviations as needed per the journal. We have defined MW and clarified all the other points in the legend.

(22) 1B: "peaks taken at a fixed interval > 5 s", this is a bit confusing. If the interval is fixed, the exact time interval should be given. If it is > 5 s, then this suggests that it is not fixed. Do you mean "at intervals > 5 s"?

Yes, fixed.

(23) Figure 1-S1C: is the area the integral of the z-scored DF/F above zero DF/F? If so, it should have units of seconds (integral over dt of a dimensionless variable). Similarly, the Peak is a z-score value? In addition, is the time to peak in seconds? What is zero? Peak time of movement?

We thank the reviewer for raising these points. We have clarified the terminology in the text and figure. Specifically, “area” was inaccurately labeled and refers to the mean z-scored ΔF/F within each analysis window (not a time integral). Peak values correspond to the maximum z-scored ΔF/F within the window, and time to peak is reported in seconds relative to the alignment point. We have also clarified the definition of time zero and included these definitions in Methods.

(24) Figure 2-S1: It is not clear if this figure is obtained by averaging across all animals. Please explain in the legend.

We clarified that values represent averages across mice.

(25) Figure 2-S2: Are the speeds in A and B in units of cm/s (vertical axis)? This needs to be indicated.

We have clarified in the figure legend that movement speed is expressed in cm/s.

(26) Figure 5A, scale bar: It looks like a Delta is missing in front of F because the label reads 0.5 F/F instead of 0.5 DF/F. I am unclear why there are three colored traces for the speed panels. If the colors denote neuron classes, does this mean they were recorded in different sessions, allowing the authors to distinguish activation speed for each class separately?

We fixed the scale bar typo. The speed traces in the bottom panels are shown to illustrate that movement is highly similar across activation types within each avoidance mode, indicating that the observed large differences in neural activity cannot be attributed to differences in movement. Minor differences in the speed traces arise because activation types are composed of neurons that can be recorded in the same or different sessions, and each activation type may not be present in every session. We added several sentences to this section that should fully clarify the issue.

(27) Figure 4-S1 legend B: Please indicate why the two panels are missing for the PA case (for the confused reader).

We have clarified in the legend that panels are not shown for correct CS2 passive avoids because these trials do not involve an action, and therefore from-action alignment cannot be defined.

(28) Figure 5-S A, B: Units missing for speed.

Fixed.

**Reviewer #3 (Recommendations for the authors):**

I cannot assess the scientific validity of the study design as it is too far away from my direct field of expertise. But I found the authors' arguments convincing, and the results sound pretty consistent with the little I know of the field. The recording methods are good and the statistical analysis robust. So my only recommendation for the authors would be to work on the figures to improve clarity.

Thank you. We have introduced various changes that we hope will facilitate readability for a wider audience while preserving the necessary details.
